# Scalable depression monitoring with smartphone speech using a multimodal benchmark and topic analysis

**DOI:** 10.1038/s41746-026-02486-9

**Published:** 2026-02-28

**Authors:** Daniel Emden, Maike Richter, Astrid Chevance, Ramona Leenings, Julian Herpertz, Lara Gutfleisch, Anna Fleuchaus, Rogério Blitz, Vincent L. Holstein, Janik Goltermann, Nils R. Winter, Jennifer Spanagel, Susanne Meinert, Tiana Borgers, Kira Flinkenflügel, Frederike Stein, Nina Alexander, Hamidreza Jamalabadi, Jonathan Repple, Christian Dobel, Elisabeth J. Leehr, Ronny Redlich, Ulrich W. Ebner-Priemer, Igor Nenadić, Tilo Kircher, Udo Dannlowski, Tim Hahn, Nils Opel

**Affiliations:** 1 Institute for Translational Psychiatry, University of Münster, Münster, Germany; 2 Department of Psychiatry and Neuroscience, Campus Benjamin Franklin, Charité-Universitätsmedizin Berlin, Berlin, Germany; 3 German Center for Mental Health (DZPG), partner site Berlin/Potsdam, Berlin, Germany; 4 Université Paris Cité and Université Sorbonne Paris Nord, INSERM INRAE, Centre for Research in Epidemiology and Statistics, Paris, France; 5 Centre d’Epidémiologie Clinique, AP-HP, Hôpital Hôtel Dieu, Paris, France; 6 Department of Psychiatry and Psychotherapy, Jena University Hospital, Jena, Germany; 7 Department of Psychiatry, Harvard Medical School, Boston, MA USA; 8 Stanley Center for Psychiatric Research, Broad Institute of MIT and Harvard, Cambridge, MA USA; 9 Institute for Translational Neuroscience, University of Münster, Münster, Germany; 10 Department of Psychiatry and Psychotherapy, Philipps-University of Marburg, Marburg, Germany; 11 Department of Psychiatry, Psychosomatic Medicine and Psychotherapy, University Hospital Frankfurt, Frankfurt am Main, Germany; 12 Cooperative Brain Imaging Center - CoBIC, Goethe University Frankfurt, Frankfurt am Main, Germany; 13 Department of Otorhinolaryngology, Institute of Phoniatry and Pedaudiology, Jena University Hospital, Jena, Germany; 14 Department of Psychology, Martin Luther University Halle-Wittenberg, Halle, Germany; 15 Center for Intervention and Research on adaptive and maladaptive brain Circuits underlying mental health (C-I-R-C), Jena-Magdeburg-Halle, Germany; 16 German Center for Mental Health (DZPG), partner site Halle-Jena-Magdeburg, Halle, Germany; 17 Mental mHealth Lab, Institute of Sports and Sports Science, Karlsruhe Institute of Technology, Karlsruhe, Germany; 18 Department of Psychiatry and Psychotherapy, Central Institute of Mental Health, University of Heidelberg, Medical Faculty Mannheim, Mannheim, Germany; 19 Center for Mind, Brain and Behavior (CMBB), Philipps-University of Marburg, Marburg, Germany; 20 Department of Psychiatry, Medical School and University Medical Center OWL, Protestant Hospital of the Bethel Foundation, Bielefeld University, Bielefeld, Germany

**Keywords:** Biomarkers, Computational biology and bioinformatics, Diseases, Health care, Medical research, Psychology, Psychology

## Abstract

Objective, scalable biomarkers are needed for continuous monitoring of major depressive disorder. Smartphone-collected speech is promising, yet clinically useful signals remain elusive. We analyzed 3151 weekly voice diaries from 284 German-speaking adults (128 MDD, 156 controls) to predict Beck Depression Inventory (BDI) scores. Sentence-embedding models outperformed lexical and acoustic baselines: Qwen3-8B achieved MAE 4.65 and *R*^2^ 0.34, and stacked generalization of multilingual-E5 with Qwen3-8B further improved performance (MAE 4.37, *R*^2^ 0.41). Audio embeddings added little incremental value. In an MDD-only analysis, multilingual-E5 was the top single modality (MAE 6.74, *R*^2^ 0.20). To aid interpretation, BERTopic uncovered six coherent themes; BDI scores were highest for “Distress & care”, supporting clinical face validity. Together, LLM embeddings paired with lightweight topic analysis capture the dominant signal of depression severity in everyday speech and offer a scalable route to ecologically valid digital phenotyping.

## Introduction

Depression is a leading contributor to the global burden of disease and is chronic and recurrent; prevention requires long-term observation, yet clinical course is typically tracked via infrequent, clinic-based self-reports^1,2^. This “snapshot” view can miss day-to-day fluctuations signaling relapse or treatment response. Digital phenotyping addresses this by continuously harvesting signals from personal devices^3,4^. Among candidate signals, spoken language is unique: it is produced spontaneously, encodes rich semantics, and carries prosodic cues linked to affect and cognition^5,6^. Voice diaries collected in *ecologically valid* contexts therefore offer a scalable, non-invasive lens on mood. Robust automatic speech recognition (ASR) is central: dialect-aware ASR has been explored for neurocognitive assessment in Alzheimer’s disease, underscoring the need to handle accent/dialect shifts in real-world patients^7^; in parallel, web-scale multilingual ASR (e.g., Whisper) shows strong zero-shot robustness^8^. These advances motivate our emphasis on ASR robustness and the effect of transcript quality/preprocessing on downstream text models.

Earlier work emphasized handcrafted acoustic markers or word-count approaches, which capture limited aspects of meaning and often generalize poorly across speakers or contexts. Transformer-based language models now provide dense sentence embeddings that compress nuanced linguistic information^9^ and are increasingly applied to mental-health analysis^10,11^. Foundational regression studies linked acoustic markers to severity in controlled settings^12,13^; scaling data and representation learning improved performance via transfer learning^14^; task-informed multimodal paradigms probed symptom dimensions beyond global severity^15^; and app-based pipelines examined uncertainty and fairness in automated speech analysis^5^. Yet few studies systematically benchmark modern text embeddings against traditional lexical/acoustic pipelines–including *self-supervised audio embeddings*–on *naturalistic, longitudinal* data.

Interpretability remains essential for clinical uptake: black-box predictions limit adoption unless researchers can articulate why a model assigns high severity^16^. Topic modeling (e.g., BERTopic^17^) can surface coherent themes in text representations and relate them to symptoms, supporting performance *and* insight–an important goal for digital phenotyping tools intended to inform therapeutic decisions rather than merely flag anomalies^18^.

Despite progress, much prior work targets MDD vs. control classification with speech elicited in controlled, laboratory settings (e.g., reading tasks, structured interviews)^19,20^. This establishes signal but not day-to-day variability within clinical populations. Recent digital phenotyping studies leverage personal devices, yet many remain cross-sectional or have not robustly modeled *longitudinal* symptom severity from speech collected over time^21,22^.

**Fig. 1:**
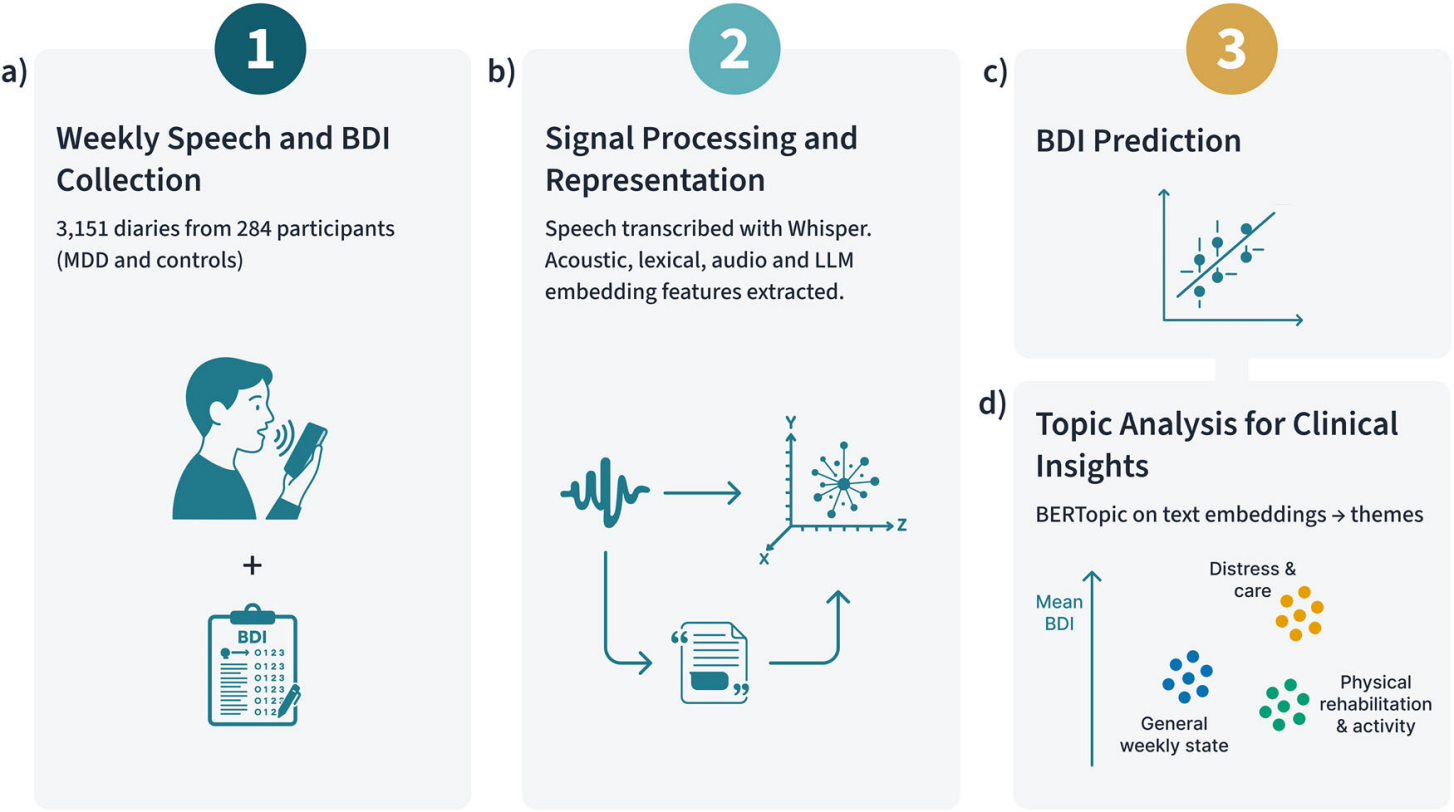
Study workflow and analysis pipeline. **a** Weekly smartphone voice diaries and Beck Depression Inventory (BDI) self-reports within ± 7 days of each diary were collected from 284 participants (MDD and HC), yielding 3151 diaries. **b** Speech was transcribed with a robust ASR system (Whisper). From transcripts and audio we derived timing/lexical features, openSMILE acoustics, self-supervised audio embeddings (e.g., Microsoft WavLM-Base, Facebook wav2vec 2.0 Base, Facebook HuBERT-Base), and LLM sentence embeddings. Solid arrows indicate the processing flow. **c** Support-vector regression predicts continuous BDI severity under participant-grouped cross-validation; the schematic scatter illustrates observed versus predicted BDI values. **d** BERTopic (topic modeling on transformer embeddings) summarizes text into interpretable themes. Colored circles denote example topic clusters (teal circles: general weekly state; gold circles: distress & care; green circles: physical rehabilitation & activity); the vertical arrow labeled “Mean BDI” indicates per-topic average severity. Abbreviations: BDI, Beck Depression Inventory; MDD, major depressive disorder; HC, healthy controls; ASR, automatic speech recognition; LLM, large language model.

Here we analyze 3151 weekly voice diaries from 284 participants, moving beyond case–control classification to model *continuous* Beck Depression Inventory (BDI) severity in a real-world clinical context. We benchmark locally runnable, open models across multimodal feature sets–timing/lexical features, TF-IDF, openSMILE acoustics, *pre-trained audio embeddings*, and *text embeddings*–within a consistent support-vector regression pipeline, and we report clinically oriented error analyses and topic-symptom links. Because transformer embeddings encode information at multiple linguistic levels–including syntax and morphology^23–25^–we complement topic modeling with *controlled linguistic perturbations* (disrupting word order, inflectional morphology, and subword regularities) to quantify their contributions. Figure 1 summarizes the six-layer analytical workflow from data collection through clinical insights.

We hypothesize that (i) *LLM sentence embeddings* (e.g., Qwen3-8B, multilingual-E5-Large-Instruct) outperform lexical and acoustic baselines for predicting continuous BDI; (ii) the resulting topic structure reveals clinically coherent themes contrasting internal distress with external activities; and (iii) perturbation sensitivity demonstrates that predictions rely on multi-level linguistic information rather than topical semantics alone. Our aim is a scalable and *partially interpretable* pipeline for continuous, voice-based depression monitoring in naturalistic settings.

## Results

### Benchmark performance across feature representations

To identify which information sources in smartphone speech most reliably track depressive symptom severity in naturalistic settings, we first benchmarked a diverse set of feature representations under a unified modeling protocol. Participant-grouped cross-validation avoids speaker leakage; permutation tests verify that gains reflect genuine structure rather than chance.

All feature-based models significantly outperformed the dummy regressor (MAE = 6.24, *R*^2^ = −0.01) in predicting continuous BDI scores (Supplementary Table 2). Label-shuffling permutation tests (1 000 iterations) confirmed that all models performed above chance (*p* < 0.001).

**Text embeddings** proved to be the strongest single modality, with large-scale sentence embeddings achieving the best performance (MAE = 4.65, *R*^2^ = 0.34). This result substantially outperformed all other baselines. The performance of smaller text embedding models and classical **TF-IDF representations** (MAE = 5.23, *R*^2^ = 0.19) was progressively worse, although TF-IDF still outperformed all acoustic and shallow lexical features.

**Audio embeddings and acoustic baselines** were less effective predictors. The best-performing audio model was audEERING wav2vec2 (MAE = 5.54, *R*^2^ = 0.06), while other models like Microsoft WavLM-Base (MAE = 5.72, *R*^2^ ≈ 0.00) and Facebook wav2vec 2.0 Base (MAE = 5.99, *R*^2^ = −0.12) showed poor performance. Shallow lexical and timing features also performed poorly (MAE = 5.83, *R*^2^ = −0.09).

Fusing multiple modalities yielded small but consistent performance gains. The most effective combination paired two text embeddings (multilingual-E5 with Qwen3-8B), which provided a modest but statistically significant improvement over Qwen3-8B alone. Text–audio fusions (e.g., with wav2vec2 or ComParE) showed less improvement and did not surpass the best text–text fusion. Hand-crafted or shallow acoustic features contributed only limited incremental signal (Table 1).

**Table 1:** Key results for BDI score prediction

Setting	Model	MAE	*R*^2^
Full cohort	**multilingual-E5 + Qwen3-8B (fusion)**	**4.37 (0.57)**	**0.41 (0.06)**
Full cohort	**Qwen3-8B (single text)**	**4.65 (0.46)**	**0.34 (0.08)**
Full cohort	Best audio-only: audEERING wav2vec2	5.54 (0.86)	0.06 (0.20)
MDD-only	**multilingual-E5 (single text)**	**6.74 (1.42)**	**0.20 (0.05)**

This table summarizes the performance of the best single-modality and fusion models in the full cohort and MDD-only regression analyses. Results are reported as Mean Absolute Error (MAE) and coefficient of determination (*R*^2^). Values in parentheses represent the standard deviation across outer cross-validation folds. Complete leaderboards and effect sizes are provided in the Supplementary Material (Supplementary Tables 2, 3, 6, and 7).

### Calibration and error analysis

**Fig. 2:**
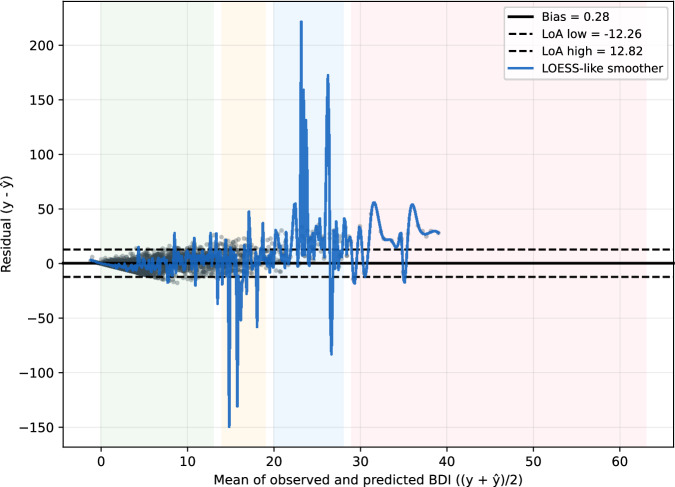
Calibration and agreement of predicted BDI scores. Bland–Altman plot for the best model (Qwen3-8B) on concatenated outer-test predictions. Residuals (observed minus predicted BDI) are plotted against the pair-wise mean (average of observed and predicted BDI). The solid black line marks the mean bias (+0.28 BDI points); dashed black lines indicate the 95% limits of agreement (−12.26 to +12.82). Shaded bands indicate BDI severity categories (0–13 minimal, 14–19 mild, 20–28 moderate, 29 or higher severe). A smooth trend line (blue line) shows proportional bias: residuals increase with symptom severity, reflecting mild underestimation at higher BDI. Abbreviation: BDI, Beck Depression Inventory.

To contextualize predictive performance, we assessed calibration and residual patterns on concatenated outer-test predictions from the best single model (Qwen3-8B). The model showed good calibration-in-the-large and a near-unity slope, indicating alignment between predicted and observed BDI scores on average. Bland–Altman analysis revealed a small mean bias with wide but clinically interpretable limits of agreement and *heteroscedastic* errors that grow with symptom severity, with a mild underestimation tendency in the severe range. While overall errors are clinically bounded (MAE ≈ 4.5), prediction error increases at higher BDI scores (Fig. 2; Supplementary Table 10). Full statistics and band-wise summaries are reported in Supplementary Result 1.

### Sensitivity analyses

To test whether models were tracking symptom variation within the clinical cohort rather than simply separating patients from controls, we repeated the regression pipeline using only data from MDD participants. In this setting, text embeddings were the only feature class with meaningful predictive power, with multilingual-E5 performing best. Full results are detailed in Supplementary Results 2 and Supplementary Tables 6 and 7.

As a complementary validation, we trained binary classifiers to distinguish MDD from HC participants. Text embeddings substantially outperformed classical lexical and acoustic baselines (see Supplementary Table 4). The best single model, Qwen3-8B, achieved a balanced accuracy of 0.71 (AUC-ROC = 0.77), and multimodal fusion offered no further improvement. All models performed significantly above chance, and effect-size analyses confirmed that embeddings were superior to baselines (see Supplementary Result 3 and Supplementary Table 5 for details).

In frequent diarists (≥10 entries), idiographic models for predicting per-person BDI change showed limited within-subject tracking: deterioration detection was near chance for thresholds of *Δ*BDI ≥3 and ≥5, with modest gains at ≥7 (see Supplementary Result 4). We therefore treat these analyses as negative but informative results and focus our main evaluation on between-person regression with careful calibration.

To probe which linguistic cues drive performance, we perturbed transcripts *before* embedding while holding the modeling pipeline fixed. All manipulations reduced performance relative to the unaltered baseline (MAE 4.56 ± 0.55, *R*^2^ = 0.325 ± 0.025). The largest degradation occurred when topical content was stripped (function-word “skeleton”), followed by word-order disruption and a content-only variant that removes function words; neutralizing inflection via lemmatization produced a smaller but reliable decline. Together, these results indicate that predictions rely on *multi-level* information: topical/lexical content contributes most, yet function-word patterns and syntax provide additional signal beyond bag-of-lemmas semantics. Full statistics are reported in Supplementary Result 5 and Supplementary Table 11.

### Topic modeling and clinical interpretation

To provide a human-readable bridge between high-dimensional embeddings and clinical symptoms, we apply BERTopic to the best-performing text embeddings to derive interpretable themes and quantify their associations with BDI scores.

**Fig. 3:**
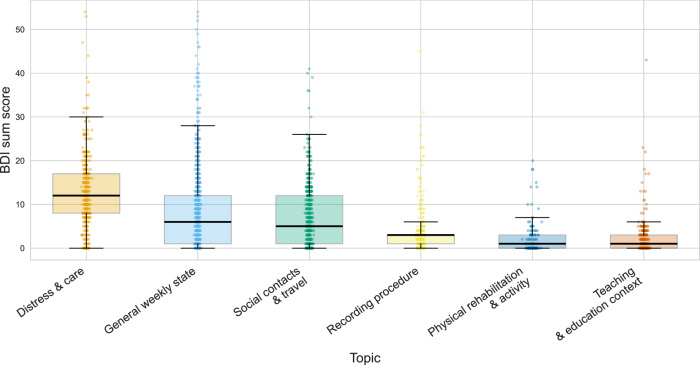
BDI severity by topic. Box-and-scatter plot of Beck Depression Inventory (BDI) sum scores by dominant theme (six themes). Each diary is assigned its dominant theme after outlier reassignment and soft-assignment fallback. Boxes show median and interquartile range (IQR); whiskers extend to 1.5 times the IQR; semi-transparent points display all diaries. Themes are ordered by mean BDI; unequal per-theme sample sizes are visible via point density. BDI, Beck Depression Inventory; IQR, interquartile range.

Using BERTopic on Qwen3-8B embeddings, we derived a reduced, clinically interpretable set of six themes (Table 2). Clustering was performed with UMAP + HDBSCAN, followed by outlier reassignment; with a soft-assignment fallback, all diaries received a dominant topic (no residual noise labels). Topics were labeled from cTF-IDF keywords and the ten most representative diaries per topic (by topic probability). Per-topic prevalence (diaries and unique participants) and BDI distributions are reported in Table 2. To visualize the underlying distributions with unequal per-topic sample sizes, we present box plots overlaid with all observations in Fig. 3.

**Table 2:** Consolidated six-theme topic summary and labeling rationale

Topic	Label rationale (Keywords & representative documents)	*n* (*N*) Mean BDI (SD)
Distress & care	Frequent self-monitoring and rumination ("*Gefühl/gefühlt*”, “*wissen nicht*”, “*Angst/Wut/Trauer*”, “*Achterbahn*”); fatigue/sleep problems; explicit care/treatment management ("Psychiater*in/Psycholog*in”, “*Selbsthilfegruppe*”, “*Klinik*”), medication decisions ("*Tabletten*”, “*Dosierung*”, “*absetzen/reduzieren*”) and coping strategies ("*Entspannungsübungen*”, “*Ablenkung*”, “*Hilfe suchen*”).	412 (40) 12.92 (8.49)
General weekly state	Standardized, formulaic weekly status updates ("*Mein Befinden in der letzten Woche*”, “*eigentlich gut*”, “*wohlgefühlt*”); focus on functional stability, positive coping, and daily activities ("*Arbeit*”, “*Garten*”, “*Sport*”, “*Urlaub*”) rather than symptom elaboration or emotional distress.	1187 (210) 8.00 (8.81)
Social contacts & travel	Social contact and mobility planning ("*Leute sehen*”, “*Besuch*”, “*Urlaub/Reisen*”) framed by external constraints and risk management ("*Maske*”, “*Abstand*”, “*Regeln/Verbote*”, “*Quarantäne*”); frustration and trade-offs (avoidance to protect others), focusing on logistics rather than inner state.	653 (131) 7.06 (6.89)
Recording procedure	Meta-commentary about completing the voice diary ("*Sprachprobe*”, app prompts such as “*zu laut*”, counting down, “*weiß nicht, was ich erzählen soll*”); procedural compliance talk, often with brief errand or travel snippets (commute/train/traffic), and minimal affective content.	438 (59) 3.48 (4.88)
Physical rehabilitation & activity	Physical rehabilitation and exercise dominate ("*laufen/schwimmen/wandern*”); injury/post-op recovery and symptom management ("*Knie/Schulter/Fuß*”, “*Orthese*”, “*Physio/Reha*”, “*Beweglichkeit*”); pacing, adapting sport routines, and functional improvement are central.	120 (26) 2.33 (3.96)
Teaching & education context	Education workplace routine and administrative load ("*Schule*”, “*Klasse*”, “*Schüler*”, “*Unterricht*”, “*Zeugnisse*”, “*Konferenzen*”); frequent bureaucratic tasks ("*Gutachten*”, “*Förderbedarf*”, “*Potenzialanalyse*”) and staffing/scheduling issues.	343 (45) 2.03 (3.92)

Topics are ordered by mean BDI. The *Stats* column displays the number of diaries (*n*), number of unique participants (*N*), and the mean BDI sum score (Standard Deviation). All diaries received a dominant topic after outlier reassignment and soft-assignment fallback.

**Fig. 4:**
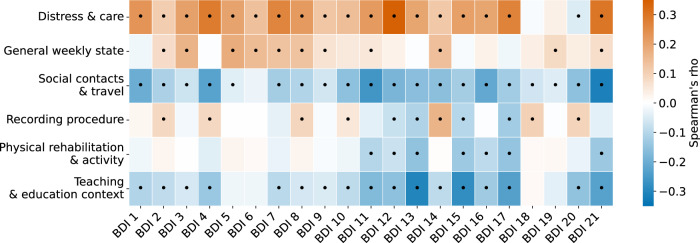
Correlations between topics and BDI items. Heat map of Spearman’s rho between the six themes and the 21 BDI items. Cells with black dots indicate Holm–Bonferroni adjusted significance (tested within each topic across items; p less than 0.05). The color scale encodes the correlation coefficient (see scale bar). BDI, Beck Depression Inventory; rho, Spearman’s rank correlation coefficient.

Because all diaries respond to the same weekly check-in prompt ("How did you feel last week?”), several themes share a common discourse framing (e.g., references to temporal markers like “last week”). We therefore distinguished topics based on their *dominant semantic focus* rather than this shared framing: for instance, *Recording procedure* captures meta-comments about the task logistics, *General weekly state* captures broad evaluative summaries of mood and functioning, and *Teaching & education context* captures specific occupational routines. Furthermore, our topic–symptom analyses utilize per-diary topic probabilities (soft assignments), which naturally accommodates diaries that may blend multiple themes (e.g., a general status update that transitions into a specific discussion of symptoms). We computed Spearman’s *ρ* between these topic probabilities and each BDI item score (Supplementary Results 6; Fig. 4).

Topics showed graded differences in BDI sum scores, with *Distress & care* associated with the highest severity and themes like *Physical rehabilitation & activity* and *Teaching & education context* with the lowest. Correlations with individual BDI items revealed clinically congruent patterns, such as *Distress & care* being linked to anhedonia and loss of interest, while activity-focused topics showed inverse associations with symptoms like fatigue and agitation.

## Discussion

We developed and evaluated a pipeline for predicting depressive symptom severity from voice diaries. Across the full cohort, sentence embeddings were the strongest single modality for BDI regression; shallow fusion with lexical/TF-IDF yielded only modest gains, and adding acoustic features contributed limited additional value. Topic modeling provided complementary interpretability: data-driven themes such as *Distress & care* and *General weekly state* associated with higher BDI scores, whereas activity-oriented themes (e.g., *Physical rehabilitation & activity*, *Teaching & education context*) aligned with lower scores, reflecting clinically coherent variation.

Prior work using free-response smartphone speech with deep topic models reports convergent, depression-relevant themes (e.g., sleep, help-seeking/therapy, work/study strain) and links longitudinal topic shifts to symptom severity, suggesting cross-context regularities in language during depressive states^26^. Complementing this, PHQ/BDI-aware language studies map text to symptom domains and, in some cases, predict BDI-II totals and item-level scores, motivating item-wise validity checks^27^. Consistent with this literature, we correlate per-diary topic probabilities with all 21 BDI items (Fig. 4), finding clinically interpretable associations (e.g., *Distress & care* with anhedonia, loss of interest, sleep disturbance). While the absolute magnitudes of these correlations are modest (*ρ* ≈ 0.15–0.30), this is characteristic of ecological monitoring, where momentary, unstructured speech behaviors are compared against retrospective, ordinal clinical scales (0–3 range). Unlike diagnostic biomarkers, natural language features in this setting are expected to yield small but directionally robust effect sizes^22^; we therefore interpret them primarily as evidence of construct validity (e.g., the directional alignment of *Physical rehabilitation & activity* with lower fatigue) rather than as standalone proxies for individual symptoms. Consequently, we position topic modeling as a post-hoc interpretability layer that contextualizes embedding-based predictions, not a surrogate for administering the BDI.

Perturbation analyses qualify what the models learn. Removing topical/lexical content (function-word skeleton) produced the largest degradation, underscoring the role of semantics; additional drops under *content-only* and *word-order disruption*, and a smaller decline under lemmatization, indicate that function-word patterns, syntax, and morphology also contribute. Performance therefore reflects *multi-level* linguistic information rather than topical content alone. In the MDD-only setting–emphasizing within-person fluctuation rather than case-control separability–*multilingual-E5* outperformed *Qwen3-8B*, while classical lexical, TF-IDF, and acoustic baselines remained weak. This dovetails with the perturbation evidence: when variation is primarily within patients over time, fine-grained linguistic cues across multiple levels–not solely topical content–are especially informative. It also suggests that, although Qwen3-8B was the most robust single modality in the full cohort, alternative embedding families can be preferable under different cohort compositions and objectives (between- vs. within-person variance).

Clinically, an out-of-sample MAE of ~ 4.5 on the 0–63 BDI scale implies predictions typically fall within one severity band. This supports *trend tracking and change detection*–for example, flagging within-person deteriorations ≥7–10 BDI points–rather than making diagnostic decisions from single estimates. Errors increase at higher severity with mild underestimation near the severe band; single predictions near cut-points warrant caution, and thresholds should prioritize sensitivity to meaningful change. Idiographic within-person *Δ*-tracking remains challenging and merits further methodological work.

Adherence was right-skewed (mean 11.1, median 2 diaries), with a long tail of frequent diarists, as is common in bring-your-own-device designs (staggered onboarding, notification fatigue, device changes, variable clinical stability). We used nested GroupKFold by participant to prevent leakage, so heavy contributors can influence model fit but not inflate test performance. The imbalance reduces within-person information for sparse contributors and may limit transportability to users who provide very few diaries. Planned robustness checks include adherence-stratified error analyses, per-participant caps or equal-weight sampling, and inverse-probability/per-subject weighting.

Topic modeling provides a post-hoc, human-readable layer that contextualizes embedding-based predictions by linking diaries to clinically coherent themes; it is not used for prediction and does not expose the internal mechanisms of the regressor. Complementary perturbation analyses indicate that the model relies on multiple linguistic levels: performance declined when topical/lexical content was removed and also under word-order disruption and lemmatization, implicating contributions from function-word patterns, syntax, and morphology. Thus, the model leverages multi-level linguistic cues rather than semantics alone. Shifts in style and structure (e.g., shorter clauses, altered grammatical marking, reduced lexical variation) can change predicted severity even when surface topics appear stable. These analyses offer behavioral evidence about information usage rather than mechanistic interpretability. Speech diaries also remain attractive for continuous, ecologically valid monitoring and, paired with topic-level summaries, can help clinicians and patients contextualize outputs. This paradigm is reflected in emerging clinical decision-support systems such as DAX Copilot (Microsoft Dragon Copilot ∣ Microsoft Cloud for Healthcare; https://www.microsoft.com/en-us/health-solutions/clinical-workflow/dragon-copilot), which could potentially improve engagement and adherence.

Generalizability and pragmatics follow from these results. Our themes were discovered in a single German-speaking cohort; topic prevalence and semantics should not be assumed universal. To strengthen transportability, future work will (i) freeze a reduced topic set on a development cohort and evaluate out-of-sample across sites, time, demographics, and languages–quantifying topic reproducibility (keyword/exemplar overlap, centroid alignment) and retention of predictive validity-and (ii) allow prospective discovery of genuinely new themes when drift is observed, followed by re-validation across old and new cohorts. For construct validity, we will test whether speech-derived topic probabilities map consistently onto intended clinical constructs by relating them to BDI item scores (convergent/discriminant patterns, known-groups differences, longitudinal sensitivity) and by checking measurement invariance across age and gender.

Several limitations apply. Outcomes relied on self-reported BDI scores; self-report bias remains possible. Our cohort (German-speaking, primarily two regions) constrains generalizability across languages, dialects, cultures, and care settings. Porting will require locale-specific validation: assess and, if needed, adapt ASR on in-domain audio; re-derive the semantic layer (topics) and re-calibrate embedding-based predictors to local language use; and perform subgroup fairness checks across dialects and demographics. Pipeline components are context-sensitive: ASR may degrade with regional accents or clinical speech phenomena; embedding geometry can shift across languages and domains; topic boundaries are corpus-dependent. Ecological variability–device microphones, sampling rates, background noise, speaking context–can induce distribution shift, motivating device-/locale-aware calibration and periodic drift monitoring. The observational design is correlational and does not establish causality; neither topic-severity associations nor perturbation sensitivity identifies mechanisms linking linguistic patterns to symptom change. Topics were derived in-sample; external replication, cross-lingual testing, and construct-validity checks against BDI items are needed before clinical use. Privacy remains central: even with local processing and derived-feature storage, speech and text can enable re-identification, so deployments should follow data-minimization and governance best practices. Finally, adherence imbalance may attenuate within-person signal for low-engagement users, and errors are heteroscedastic, increasing with severity.

In sum, modern LLM sentence embeddings provided the strongest single-modality basis for regressing depressive symptom severity from naturalistic speech; shallow fusion added only modest gains, and acoustic features contributed limited additional value in this setting. In MDD-only analyses, multilingual-E5 surpassed Qwen3-8B, underscoring that the optimal embedding family can depend on cohort composition and within-person objectives. Controlled perturbations demonstrated reliance on multiple linguistic levels–topical/lexical content, word-order/syntax, and, to a lesser extent, inflectional morphology–rather than semantics alone. Topic modeling offers a practical, human-readable lens; paired with calibration, change-sensitive thresholds, and careful validation in new populations, this approach supports continuous, speech-based monitoring of depressive symptoms in real-world contexts.

## Methods

### Cohort and data acquisition

Data were drawn from a longitudinal project using the ReMAP (Remote Monitoring Application in Psychiatry) smartphone app to acquire weekly speech samples from healthy controls (HC) and individuals with a lifetime or current diagnosis of Major Depressive Disorder (MDD). The full dataset comprises 3,151 weekly speech samples from 284 unique participants (HC: 156; MDD: 128), collected between May 2019 and April 2025. Participants were recruited primarily from ongoing mental-health cohort studies, with more than two-thirds originating from the Marburg-Münster Affective Disorders Cohort Study (MACS) at two sites (Marburg and Münster, Germany) under identical protocols^28,29^. The presence or absence of a lifetime mental disorder was confirmed via the Structured Clinical Interview for DSM-IV (SCID-IV) within the source cohorts^30^. The MDD group included both acutely depressed individuals and those in remission, spanning a broad range of symptom severity. Following informed consent and a bring-your-own-device approach, participants installed ReMAP on personal iOS/Android smartphones. For voice sampling, the app issued randomized daytime prompts once per week; participants responded by speaking freely for 1–3 minutes to the German question: *"Wie haben Sie sich in der letzten Woche gefühlt?”* Technical details of the ReMAP platform have been reported previously^31,32^.

Demographic and clinical characteristics are summarized in Table 3. Across the observation window, participants contributed a mean of 11.1 samples (SD = 28.5), with high between-person variability (median = 2, IQR = 1–7; maximum = 282). The mean per-participant follow-up was 151 days (median = 17; IQR = 0–137).

**Table 3:** Demographic and clinical characteristics of the study sample and group differences

Characteristic	Healthy Controls	MDD	Total	Statistic
No. of Participants, *N* (%)	156 (54.9%)	128 (45.1%)	284 (100.0%)	
No. of Samples (*n*)	1880	1271	3151	
Age, years [Mean (SD)]	53.04 (11.73)	49.69 (11.45)	51.70 (11.73)	t = −7.31, *p* < 0.001
Sex, *N* (%)				*χ*^2^ = 65.28, *p* < 0.001
Female	107 (37.7%)	93 (32.7%)	200 (70.4%)	
Male	49 (17.3%)	35 (12.3%)	84 (29.6%)	
BDI Score at Time of Sample [Mean (SD)]	3.47 (4.29)	12.14 (9.36)	6.97 (8.03)	t = −30.90, *p* < 0.001

*MDD* Major Depressive Disorder, *BDI* Beck Depression Inventory, *SD* Standard Deviation. *P*-values from independent t-tests for continuous variables and Chi-squared tests for categorical variables.

The primary outcome was depressive-symptom severity measured with the Beck Depression Inventory (BDI), administered in-app once weekly. For each speech sample, we paired the temporally closest BDI completed within a ± 7-day window; this pairing rule was pre-specified and applied uniformly to all analyses. Thus, each diary entry is associated with a proximate self-report capturing within-person symptom fluctuations over time.

All procedures complied with the Declaration of Helsinki and were approved by the local ethics committees at both study sites (Marburg and Münster). Participants provided written informed consent prior to enrollment, including consent for app-based data collection and secondary analyses of de-identified data. The study design, recruitment, and feasibility of smartphone-based assessments via ReMAP are detailed elsewhere^31,32^.

All audio diaries were transcribed *locally* with the open-source Whisper large-v2 model^8^, using German ASR with automatic punctuation and casing enabled. We retained the verbatim output, including disfluencies, fillers, and function words. Local execution was chosen to reduce data movement; however, local processing does not itself guarantee privacy.

For downstream modeling, each diary yielded two parallel transcript variants. First, a verbatim transcript was kept exactly as produced by the ASR system. Second, a normalized variant was derived with a standard German NLP pipeline (lowercasing, removal of punctuation and stop words, and lemmatization). We computed all contextual text embeddings from the verbatim transcripts to preserve surface-form cues–such as function-word usage, negation markers, tense/aspect indicators, and disfluencies–that may carry clinically informative signal. In contrast, sparse lexical features (TF-IDF) and topic keyword extraction used the normalized text to consolidate vocabulary and reduce sparsity. Samples with empty or failed transcripts were excluded from text-based analyses.

Regarding transcription accuracy, we did not conduct a manual validation or human annotation study. Instead, we relied on Whisper large-v2’s published performance, which reports low word-error rates for German on standard benchmarks^8^. We acknowledge that residual ASR errors and environmental noise can affect lexical content and, in turn, downstream features; this potential source of measurement error is noted in the Limitations.

### Feature representations and models

We computed sentence-level (utterance-level) embeddings on verbatim transcripts (no lowercasing, lemmatization, or stopword removal) using six open models runnable locally: Qwen3-Embedding-8B (Qwen/Qwen3-Embedding-8B), Qwen3-Embedding-4B (Qwen/Qwen3-Embedding-4B), Qwen3-Embedding-0.6B (Qwen/Qwen3-Embedding-0.6B)^33^, EmbeddingGemma-300M (google/embeddinggemma-300m^34^), multilingual-E5-Large-Instruct (intfloat/multilingual-e5-large-instruct^35^), and All-MPNet-Base-v2 (sentence-transformers/all-mpnet-base-v2^36^). We used each model’s default sentence representation to obtain one fixed-length vector per transcript.

From mono 16 kHz audio, we computed two standard paralinguistic sets with the Python openSMILE toolkit: ComParE 2016 functionals (6,373 summary statistics over prosodic, spectral, and voice-quality LLDs;^37^) and eGeMAPS v02 (88 psychologically motivated descriptors;^38^). Both summarize frame-level descriptors per recording into fixed-length vectors.

We extracted fixed-length representations from four pretrained self-supervised speech models (Hugging Face Transformers): WavLM-Base (microsoft/wavlm-base)^39^, HuBERT-Base (facebook/hubert-base-ls960)^40^, wav2vec 2.0 Base (facebook/wav2vec2-base)^41^, and wav2vec2-Large-Robust (12-layer) fine-tuned for dimensional emotion on MSP-Podcast (audeering/wav2vec2-large-robust-12-ft-emotion-msp-dim)^42^. All audio was converted to mono 16 kHz and capped at 60 s. For each model, we mean-pooled the last hidden layer across time using the attention mask to obtain a single embedding per recording. For the audEERING wav2vec2 model, we additionally extracted dimensional emotion scores (arousal, dominance, valence) and concatenated them to the embedding in downstream analyses.

A set of 18 timing and lexical statistics computed from transcripts and voice activity detection (Supplementary Table 1). Speech and pause durations were extracted using ffmpeg silence detection (threshold: −30 dB, minimum silence duration: 0.5 s). Transcripts were processed with spaCy (de_core_news_md) for tokenization, POS tagging, and named entity recognition.

Term Frequency-Inverse Document Frequency vectors from 1 to 2 gram features on normalized transcripts (standard preprocessing; see Preprocessing), weighting terms by corpus rarity to highlight diary-specific words/phrases.

We prioritized *open* audio and text encoders that can be executed locally to reduce data movement; however, local execution does not by itself ensure privacy. Models were selected if they are widely used in clinical/digital-phenotyping studies^5,21,43^ or competitive on public embedding benchmarks (MTEB (https://huggingface.co/spaces/mteb/leaderboard; accessed Oct. 1, 2025)).

Multimodal combinations were pre-specified as fixed two-way pairings to test complementarity with text embeddings. For each of the two best-performing text-embedding models (selected on the full cohort), we paired them with timing & lexical features (TLF), TF-IDF, openSMILE ComParE, openSMILE eGeMAPS, the best audio-embedding model, and the other top text-embedding model. Pairings were defined on the full cohort and then applied unchanged to the MDD-only and HC-only subgroups.

### Evaluation and statistical analysis

For each feature set we trained a scikit-learn pipeline with z-score standardization, PCA, and an SVR to predict Beck Depression Inventory (BDI) scores recorded within ± 7 days of each diary. We evaluated every *Single Modality* and predefined *Multimodal Combination*. Multimodal models used late fusion (stacked generalization): modality-specific base models were fit within the inner loop and their out-of-fold predictions fed a Ridge meta-learner, which was refit on the full outer-training split and evaluated on the held-out fold.

Performance was estimated with nested, participant-stratified cross-validation (five outer folds, three inner folds; GroupKFold with participant ID as group). Identical outer folds were used across all models. Preprocessing and fitting were learned on training partitions only and applied to test data via fitted transformers. Because participation was highly imbalanced (many participants contributed 1–2 diaries, while a minority contributed dozens), we used nested GroupKFold with the participant ID as the grouping variable to prevent subject-level leakage.

For regression we report outer-fold mean absolute error (MAE) and explained variance (*R*^2^). For the HC vs. MDD sensitivity analysis we report balanced accuracy and AUROC. Unless stated otherwise, results are mean ± SD across outer folds and, for stacked models, computed from the meta-learner’s outer-fold predictions.

All significance testing used outer-fold predictions. Head-to-head comparisons used paired sign-flip permutation tests over folds with small-sample correction; we report two-sided *p*-values, mean paired differences with bootstrap 95% CIs, and paired Cohen’s *d*_*z*_ (Cliff’s *Δ* additionally for MAE). For contrasts between multimodal and corresponding single-modality models on *R*^2^, we also summarise Cohen’s *f*^2^ with bootstrap CIs.

On concatenated outer-test predictions we estimated calibration-in-the-large (intercept) and calibration slope by regressing *y* on $\widehat{y}$, reporting coefficients with bootstrap 95% CIs. Agreement was further assessed via Bland–Altman analysis (bias and 95% limits of agreement with bootstrap CIs) and a regression of residuals on means to check proportional bias.

Inner-loop hyperparameters were tuned via a randomized cross-validated search with a fixed budget per outer fold and a reproducible random state. We searched over the SVR kernel, *C*, *ϵ*, and PCA dimensionality; the selected configuration was refit on the full outer-training split and evaluated once on the outer-test split.

We included simple baseline models as reference points: a mean-prediction baseline for regression and a stratified baseline reflecting class prevalence for the HC vs. MDD sensitivity analysis.

Analyses used Python 3.11 and scikit-learn 1.7 with fixed random seeds and fold indices.

To contextualize the main regression results and test the models’ robustness, we conducted four additional analyses. All procedures followed the same nested cross-validation protocol as the main analysis unless otherwise noted.

To ensure the models were tracking symptom variation within the clinical cohort rather than simply distinguishing patients from controls, we repeated the full regression pipeline using only data from participants with MDD.

For completeness, we also repeated the regression pipeline using only data from the healthy control (HC) cohort. This analysis serves as a negative control, as we would not expect the models to predict meaningful variance in BDI scores within a non-clinical population where scores are uniformly low and show little variation.

As a complementary validation of the feature sets, we trained binary classifiers to distinguish participants with MDD from healthy controls (HC), reporting balanced accuracy and AUROC.

To evaluate the models for longitudinal monitoring, we tested their ability to predict weekly *changes* in BDI scores (*Δy*_*t*_ = *y*_*t*_ − *y*_*t*−1_) for individual participants. For frequent diarists (≥10 entries), we trained person-specific (idiographic) models using Qwen3-8B embeddings to predict *Δy*_*t*_. We evaluated performance by comparing per-subject correlations and Mean Absolute Error (MAE) against several baselines, including a no-change model and a calibrated global model. We also assessed the models’ accuracy in detecting clinically meaningful deteriorations at various thresholds (*Δ*BDI ≥ 3, 5, and 7).

### Linguistic perturbation procedures

Embeddings from large language models (LLMs) can encode linguistic information at multiple levels beyond topical/semantic content, including syntactic relations, inflectional morphology, and subword/orthographic regularities. To test which of these levels contribute to prediction in our setting, we applied three controlled text perturbations prior to embedding. Each manipulation selectively disrupts a targeted property while holding others as constant as possible. We then recomputed embeddings and re-evaluated the identical regression pipeline to quantify the effect of each perturbation on performance.

For each condition, we (i) transformed the verbatim German transcript, (ii) recomputed Qwen3-8B sentence embeddings, and (iii) re-ran the *same* PCA → SVR pipeline under nested, participant-stratified five-fold cross-validation (GroupKFold in outer and inner loops) with inner-loop hyperparameter tuning over the baseline search space. Performance is summarized as mean absolute error (MAE) and explained variance (*R*^2^) averaged across outer folds. Pairwise differences to the unperturbed baseline are assessed with paired permutation tests (1000 iterations within outer folds); we also report Cohen’s *d* for MAE. Random seeds, fold assignments, and preprocessing settings are fixed across conditions. Baseline embedding/pipeline details are otherwise unchanged.

- Word-order disruption (syntactic ablation): To disrupt syntactic structure while preserving lexical content, we randomly permute token order *within* each sentence (one uniformly random permutation per sentence). Tokens are neither added nor removed; sentence boundaries are preserved; punctuation is reattached by simple heuristics. The permuted text is re-embedded and evaluated with the identical nested-CV protocol. A performance decline here is interpreted as reliance on word order and syntactic relations in the embeddings.
- Inflection neutralization (morphology ablation): To suppress inflectional morphology while preserving coarse lexical semantics, we replace each token with its lemma using a German NLP pipeline (POS-aware; numerals and proper nouns left unchanged). The lemmatized transcript is re-embedded and evaluated as above. A selective decline indicates that morphological cues (e.g., tense, case, number, gender) contribute signal beyond bag-of-lemmas semantics.
- Subword noise injection (orthographic/phonotactic proxy): As lightweight proxies for subword and phonotactic information, we apply two character-level manipulations prior to embedding: (i) *vowel masking* (German vowels {a, e, i, o, u, ä, ö, ü} replaced by “_”), and (ii) *intra-word shuffling* that preserves the first and last character but randomly permutes interior characters for tokens of length ≥5. Both variants are re-embedded and evaluated under the same protocol. Sensitivity here suggests that subword/orthographic regularities (which correlate with phonotactics) contribute to predictive performance.

All transforms operate on UTF-8 verbatim transcripts with sentence tokenization via spaCy/Stanza. For each condition, we recompute Qwen3-8B embeddings in batches on GPU. Within the inner loop, the SVR kernel, regularization parameter *C*, *ϵ*-insensitive width, and the number of retained PCA components are re-tuned using the identical search space and early-stopping criteria as the baseline. We log fold-wise predictions to enable paired testing and effect-size estimation.

### Topic modeling procedures

To characterize linguistic themes associated with depression severity, we conducted unsupervised topic modeling with BERTopic^17^ using the best-performing text-embedding model (Qwen3-8B). Embeddings were computed *once* on raw transcripts and kept fixed for all topic analyses. For label interpretability, we derived an additional cleaned textual representation (German lemmatisation, stop-word removal, content-word filtering) used solely by BERTopic to extract concise keywords; clustering operated in the embedding space.

We applied UMAP for dimensionality reduction followed by HDBSCAN for density-based clustering. HDBSCAN noise points (label −1) were handled via *outlier reassignment* to the nearest cluster in embedding space; when ambiguity remained, a *soft-assignment fallback* based on per-document topic probabilities assigned the highest-probability topic. Topics were labeled by jointly inspecting BERTopic’s cTF-IDF keywords and the ten most representative transcripts per topic (highest topic probability).

Semantically overlapping clusters were merged into a consolidated six-theme set aimed at clinical interpretability. This set was used for all summaries and statistical analyses. For descriptive reporting, each diary was assigned its *dominant* topic. Entries that could not be confidently reassigned from the noise cluster were excluded from summaries but retained for diagnostics.

For each topic, we report the number of diaries and unique participants and the mean (SD) of the BDI sum score. Between-topic differences in BDI were assessed with a Kruskal–Wallis *H*-test (primary omnibus). We additionally correlated per-recording topic probabilities with BDI item scores using Spearman’s *ρ* with 95% CIs via Fisher *z*, controlling the familywise error rate within the 21 items per topic using Holm–Bonferroni. All tests were two-sided, with diary as the unit of analysis; per-topic summaries additionally report the number of unique participants to contextualize repeated measures.

## Supplementary information

Supplementary information

## Data Availability

The raw audio recordings and verbatim transcripts contain potentially re-identifiable health information and were collected under multiple study protocols approved by local ethics committees. They cannot be deposited in a public repository. De-identified feature matrices (acoustic, lexical, TF-IDF and Qwen3-8B embeddings) together with the training and evaluation code are available from the corresponding author on reasonable request. Access will require a data-use agreement and approval from the principal investigators of the contributing studies.
