# Supplementary material for: Scalable depression monitoring with smartphone speech using a multimodal benchmark and topic analysis
Source: NPJ Digit Med. 2026 Feb 28;9:230. doi: 10.1038/s41746-026-02486-9 (PMC12996298; doi:10.1038/s41746-026-02486-9)
Supplement: Supplementary file 1 — Supplementary information [file 41746_2026_2486_MOESM1_ESM.pdf]

Supplementary Material

Supplementary Result 1. Calibration and error analysis

We evaluated calibration and residual structure using concatenated outer-test predictions from Qwen3-8B. Calibration-in-the-large was good (intercept = 0.37; 95% CI: [0.03, 0.71]) with a near-unity slope (0.99; [0.93, 1.04]). Bland–Altman analysis showed a small mean bias of +0.28 BDI points ([0.06, 0.50]) and 95% limits of agreement spanning −12.26 to +12.82 (Fig. 2). A regression of residuals on the pairwise mean indicated significant proportional bias ($\beta_1 = 0.59$; [0.54, 0.65]; $p < 0.001$), consistent with increasing error magnitude at higher severity and a mild underestimation in the severe range. Outliers beyond the limits of agreement comprised 160 diaries (5.1%) from 55 participants; trimming the top 1% of absolute errors changed MAE from 4.52 to 4.27, indicating errors were not driven by a few extreme cases.

In summary, calibration is adequate on average with low mean bias; errors are heteroscedastic, increasing with BDI severity and showing a slight underestimation tendency at the high end. These properties support use cases emphasizing *within-person trend tracking and change detection* (e.g., thresholds ≥7–10 points) rather than single-shot diagnostic decisions.

Supplementary Result 2. MDD-Only Regression

To test whether our models were simply separating healthy controls from patients rather than tracking symptom variation within a clinical cohort, we repeated the entire predictive-modeling pipeline using only the 1,271 diaries contributed by the 128 MDD participants.

In this more challenging setting, text embeddings remained the only feature class to demonstrate meaningful predictive power. multilingual-E5 achieved the best single-modality performance (MAE = 6.74, $R^2 = 0.20$), closely followed by Qwen3-8B (MAE = 6.86, $R^2 = 0.17$) and Qwen3-4B (MAE = 6.93, $R^2 = 0.16$). In contrast, all other feature modalities performed at or below the dummy baseline (MAE = 7.37, $R^2 = -0.06$): TF–

IDF achieved only marginal predictive power (MAE = 6.98, $R^2 = 0.10$), while timing & lexical features (MAE = 7.71, $R^2 = -0.13$) and all acoustic features (R^2 ranging from -0.06 to 0.02) showed no meaningful signal. Multimodal fusion provided modest gains, with multilingual-E5 + audEERING wav2vec2 achieving the best overall performance (MAE = 6.08, $R^2 = 0.22$). Effect-size analyses (Table 7) confirmed that Qwen3-8B significantly outperformed classical and acoustic baselines (Cohen's d_z ranging from -1.67 to -2.93, Cliff's $\Delta = -1.00$), though the difference from TF–IDF was not statistically significant ($\Delta R^2 = 0.07$, 95% CI: -0.16 to 0.03).

Supplementary Result 3. HC vs. MDD Classification

We trained binary classifiers to distinguish participants with MDD from HCs, using the same nested cross-validation protocol as the main regression analysis. We report balanced accuracy and AUROC.

Qwen3-8B embeddings alone achieved a balanced accuracy of 0.71 (AUC-ROC = 0.77), markedly surpassing the strongest classical baseline (TF–IDF: 0.64 balanced accuracy, AUC-ROC = 0.70) and all acoustic-only models (best: audEERING wav2vec2, 0.58 balanced accuracy). Among text embeddings, performance scaled with model size: Qwen3-4B (0.69), Qwen3-0.6B (0.69), and multilingual-E5 (0.68) all exceeded classical baselines, while smaller models (EmbeddingGemma: 0.63, All-MPNet-Base-v2: 0.60) showed weaker discrimination. The best multimodal combination (multilingual-E5 + Qwen3-8B) matched the performance of Qwen3-8B alone (0.71 balanced accuracy, AUC-ROC = 0.79). All other fusion combinations showed small declines in bACC/AUROC relative to their stronger constituent modality, consistent with late fusion adding variance when the second modality contributes minimal incremental signal. Effect-size analyses (Table 5) revealed no significant difference between the top-performing embedding model and the multimodal combination ($\Delta = 0.00$, Cohen's $d_z = 0.06$), while embeddings significantly outperformed TF–IDF ($\Delta = 0.07$, Cohen's $d_z = -0.91$, Cliff's $\Delta = -0.60$) and all acoustic features (Cohen's d_z ranging from –0.90 to –1.19, Cliff's $\Delta = -0.60$ to –1.00). Label-shuffling permutation tests confirmed that every classification model performed significantly above

chance ($p < 0.001$).

Supplementary Result 4. Within-subject change prediction (ΔBDI)

For participants with ≥ 10 diary–BDI pairs, we derived consecutive-score changes per person ($\Delta\text{BDI}_t = \text{BDI}_t - \text{BDI}_{t-1}$). We trained idiographic models (one per participant) using the same feature engineering and grouped cross-validation, evaluating: (i) regression on ΔBDI (MAE, RMSE), and (ii) deterioration detection at thresholds $\Delta\text{BDI} \geq \{3,5,7\}$ (balanced accuracy, AUROC). Speech–BDI pairs followed the same proximity rule as the main analysis.

Across frequent diarists, within-person Δ regression showed near-zero tracking, and deterioration detection was near chance for $\Delta\text{BDI} \geq 3$ and ≥ 5, with modest improvement at ≥ 7. Results were consistent under alternative splits and remained qualitatively similar when restricting to MDD-only.

Several factors plausibly limit idiographic Δ performance: (1) *Low SNR of differences*: differencing two noisy self-reports (and allowing timing jitter) amplifies measurement error; (2) *Event scarcity*: large deteriorations are rare per person, yielding class imbalance and unstable thresholds; (3) *Small per-subject n*: even with ≥ 10 entries, train/test partitions are shallow, increasing variance and overfitting risk; (4) *Irregular sampling*: variable gaps and autocorrelation complicate change modeling.

These negative results argue for pooled models with *partial pooling* rather than fully idiographic fits (e.g., hierarchical/mixed-effects or subject-specific offsets), personalized calibration, and denser, better-aligned BDI–speech pairing. We consider change-point detection and Bayesian state-space formulations promising for prospective monitoring.

Supplementary Result 5. Linguistic perturbation analyses

We applied controlled transcript perturbations to attribute performance changes to specific linguistic levels while keeping the downstream pipeline fixed. Baseline uses unaltered transcripts. The following variants were tested: (i) word-order disruption (permute token order within sentences; syntax ablation), (ii) lemmatization (neutralize inflection; mor-

phology ablation), (iii) function-word skeleton (remove content words; retain function words and inject subword noise), and (iv) content-only (remove function words; retain content words under subword noise). Paired permutation tests (within outer-fold out-of-sample predictions; $n = 3165$ pairs) compared each variant to baseline.

Effect sizes (Cohen's d on absolute errors) were small-to-moderate: word-order $d = 0.123$, lemmatization $d = 0.032$, function-word skeleton $d = 0.217$, content-only $d = 0.123$. Collectively, the pattern supports a multi-level account in which topical/lexical content is primary, with measurable contributions from function-word distributions and syntactic order; purely lemma-level content does not fully recover baseline performance.

Supplementary Result 6. Topic-BDI Associations

We expected topic–item correlations to be modest because (i) individual BDI items have a restricted ordinal range (0–3), (ii) free-response diaries need not verbalize specific symptoms even when they are present, and (iii) topics capture broad contexts rather than item-specific symptom expressions. We therefore interpret Spearman's ρ primarily as a directional convergent/discriminant validity check, and we emphasize topic differences in BDI sum-score distributions as the main interpretability result.

Topics showed graded differences in BDI sum scores, with *Distress & care* associated with the highest severity and themes like *Physical rehabilitation & activity* and *Teaching & education context* with the lowest. Correlations with individual BDI items revealed clinically congruent patterns, such as *Distress & care* being linked to anhedonia and loss of interest, while activity-focused topics showed inverse associations with symptoms like fatigue and agitation. Full details are provided in Supplementary Results 6 and Fig. 4.

To further assess the differential association of topis with specific depressive symptoms, we correlated per-recording topic probabilities with BDI item scores (Spearman's ρ with 95% CIs via Fisher z), controlling the familywise error rate within the 21 items per topic using Holm–Bonferroni. Several theoretically concordant associations emerged, for example: *Distress & care* with loss of interest (BDI 12; $\rho \approx 0.32$), loss of interest in sex (BDI 21; $\rho \approx 0.29$), anhedonia (BDI 4; $\rho \approx 0.27$), and sleep changes (BDI 16;

$\rho \approx 0.18$); *Recording procedure* inversely with agitation (BDI 11; $\rho \approx -0.25$), anhedonia (BDI 4; $\rho \approx -0.22$), and loss of interest in sex (BDI 21; $\rho \approx -0.30$); *Physical rehabilitation & activity* inversely with energy/fatigue (BDI 15; $\rho \approx -0.12$), irritability (BDI 17; $\rho \approx -0.13$), and loss of interest in sex (BDI 21; $\rho \approx -0.13$); and *Teaching & education context* inversely with indecisiveness (BDI 13; $\rho \approx -0.28$), loss of interest (BDI 12; $\rho \approx -0.15$), and irritability (BDI 17; $\rho \approx -0.22$).

Supplementary Table 1: Summary of timing and lexical features extracted from speech transcripts and audio recordings. Timing & Lexical Features (TLF) were extracted from speech transcripts and audio recordings. Timing features were extracted using `ffmpeg` silence detection (-30 dB threshold, 0.5 s minimum silence). Lexical and linguistic features were computed using `spaCy` (`de_core_news_md`) tokenization, POS tagging, and NER on German transcripts.

**Feature**	**Description**
Timing	
duration_seconds	Total duration of the recording.
speak_seconds	Total duration of detected speech.
speech_ratio	Proportion of the recording that is speech.
pause_seconds	Total duration of pauses.
pause_ratio	Proportion of the recording that is pauses.
pause_to_speech_ratio	Pause duration relative to speech duration.
words_per_total_second	Speech rate over total duration.
words_per_speak_second	Articulation rate over speech-only time.
Lexical counts / composition	
word_count	Total words spoken.
unique_word_count	Number of unique word types.
lexical_diversity	Type–token ratio (unique / total words).
char_length	Total characters in transcript.
avg_word_length	Average word length (characters).
Linguistic ratios (NER / POS)	
entity_count	Number of named entities.
entity_density	Named entities per word.
noun_ratio	Proportion of tokens that are nouns.
verb_ratio	Proportion of tokens that are verbs.
adj_ratio	Proportion of tokens that are adjectives.

Abbreviations: TLF, Timing & Lexical Features; POS, part-of-speech; NER, named entity recognition.

Supplementary Table 2: Predictive performance of feature sets for BDI score regression (full cohort). All models are Support Vector Regressors with hyperparameters tuned via 5×3 nested cross-validation (5 outer, 3 inner folds). Best single modality and best multimodal combination are bold.

Feature Set	k (features)	MAE (SD)	R^2 (SD)
Any Feature Set (Dummy Baseline)	—	6.24 (0.35)	-0.01 (0.05)
**Single Modality**			
**Qwen3-8B**	**4096**	**4.65 (0.46)**	**0.34 (0.08)**
multilingual-E5	1024	4.68 (0.58)	0.34 (0.12)
Qwen3-4B	2560	4.82 (0.47)	0.32 (0.06)
Qwen3-0.6B	1024	4.98 (0.40)	0.25 (0.06)
TF–IDF	variable	5.23 (0.57)	0.19 (0.05)
EmbeddingGemma	768	5.13 (0.50)	0.17 (0.06)
All-MPNet-Base-v2	768	5.53 (0.44)	0.10 (0.04)
audEERING wav2vec2	1027	5.54 (0.86)	0.06 (0.20)
Microsoft WavLM-Base	768	5.72 (0.71)	0.00 (0.12)
Facebook wav2vec 2.0 Base	768	5.99 (0.82)	-0.12 (0.16)
Timing & Lexical features	18	5.83 (0.67)	-0.09 (0.15)
eGeMAPS	88	6.11 (0.68)	-0.09 (0.07)
ComParE	6373	6.04 (0.69)	-0.06 (0.08)
Facebook HuBERT-Base	768	5.99 (0.58)	-0.04 (0.13)
**Multimodal Combinations**			
**multilingual-E5 + Qwen3-8B**	**4096 + 1024**	**4.37 (0.57)**	**0.41 (0.06)**
Qwen3-8B + audEERING wav2vec2	4096 + 1027	4.35 (0.42)	0.38 (0.10)
Qwen3-8B + ComParE	4096 + 6373	4.43 (0.42)	0.38 (0.10)
Qwen3-8B + TF–IDF	4096 + variable	4.44 (0.41)	0.37 (0.10)
Qwen3-8B + Timing & Lexical features	4096 + 18	4.45 (0.36)	0.37 (0.12)
Qwen3-8B + eGeMAPS	4096 + 88	4.45 (0.36)	0.36 (0.10)
multilingual-E5 + audEERING wav2vec2	1024 + 1027	4.45 (0.38)	0.36 (0.13)
multilingual-E5 + ComParE	1024 + 6373	4.54 (0.50)	0.36 (0.12)
multilingual-E5 + TF–IDF	1024 + variable	4.55 (0.48)	0.35 (0.11)
multilingual-E5 + Timing & Lexical features	1024 + 18	4.56 (0.46)	0.35 (0.12)
multilingual-E5 + eGeMAPS	1024 + 88	4.58 (0.42)	0.34 (0.11)

Abbreviations: MAE, mean absolute error; R^2, coefficient of determination; SD, standard deviation.

Supplementary Table 3: Pairwise effect size comparisons between models for the main regression analysis. Effect sizes are based on R^2. Δ is defined as B−A (Feature Set B minus A); higher is better, so negative Δ favors the first model (A). Cohen's d_z is computed on outer-fold differences; 95% CIs from 10,000 bootstrap iterations.

**Comparison**	**ΔR^2 (95% CI)**	**Cohen's d_z (95% CI)**	**Cliff's Δ**
Qwen3-8B vs			
TF–IDF	-0.15 (-0.19, -0.11)	-3.03 (-8.41, -2.41)	-1.00
Timing & Lexical	-0.44 (-0.59, -0.31)	-2.40 (-15.84, -2.06)	-1.00
audEERING wav2vec2	-0.29 (-0.40, -0.17)	-1.87 (-7.45, -1.60)	-1.00
eGeMAPS	-0.44 (-0.50, -0.36)	-4.67 (-34.13, -3.46)	-1.00
ComParE	-0.41 (-0.48, -0.31)	-3.58 (-15.48, -2.39)	-1.00
multilingual-E5 + Qwen3-8B	0.07 (-0.01, 0.17)	0.56 (-0.30, 1.50)	0.60
Qwen3-8B + audEERING	0.04 (-0.06, 0.14)	0.30 (-0.67, 2.14)	0.20

Abbreviations: CI, confidence interval; Δ, difference (B minus A); R^2, coefficient of determination; d_z, Cohen's d_z.

Supplementary Table 4: Classification performance for distinguishing participants with major depressive disorder from healthy controls. All models are Support Vector Classifiers with hyperparameters tuned via 5×3 nested cross-validation (5 outer, 3 inner folds). Best single modality and best multimodal combination are bold.

Feature Set	k (features)	bACC (SD)	AUC-ROC (SD)
Any Feature Set (Dummy Baseline)	—	0.50 (0.00)	0.50 (0.00)
**Single Modality**			
**Qwen3-8B**	**4096**	**0.71 (0.10)**	**0.77 (0.13)**
Qwen3-4B	2560	0.69 (0.07)	0.75 (0.09)
Qwen3-0.6B	1024	0.69 (0.08)	0.75 (0.11)
multilingual-E5	1024	0.68 (0.07)	0.73 (0.11)
TF–IDF	variable	0.64 (0.02)	0.70 (0.05)
EmbeddingGemma	768	0.63 (0.04)	0.68 (0.04)
Timing & Lexical features	18	0.61 (0.04)	0.67 (0.06)
All-MPNet-Base-v2	768	0.60 (0.04)	0.65 (0.08)
Facebook HuBERT-Base	768	0.59 (0.11)	0.60 (0.15)
Microsoft WavLM-Base	768	0.58 (0.10)	0.62 (0.12)
audEERING wav2vec2	1027	0.58 (0.06)	0.63 (0.05)
ComParE	6373	0.57 (0.11)	0.61 (0.13)
eGeMAPS	88	0.55 (0.08)	0.56 (0.07)
Facebook wav2vec 2.0 Base	768	0.52 (0.04)	0.55 (0.05)
**Multimodal Combinations**			
**multilingual-E5 + Qwen3-8B**	**4096 + 1024**	**0.71 (0.06)**	**0.79 (0.09)**
Qwen3-8B + TF–IDF	4096 + variable	0.69 (0.09)	0.76 (0.12)
Qwen3-8B + eGeMAPS	4096 + 88	0.69 (0.08)	0.75 (0.12)
Qwen3-8B + Timing & Lexical features	4096 + 18	0.68 (0.09)	0.75 (0.13)
Qwen3-8B + ComParE	4096 + 6373	0.68 (0.09)	0.74 (0.13)
Qwen3-8B + audEERING wav2vec2	4096 + 1027	0.68 (0.06)	0.76 (0.10)
multilingual-E5 + Timing & Lexical features	1024 + 18	0.65 (0.07)	0.71 (0.11)
multilingual-E5 + TF–IDF	1024 + variable	0.64 (0.06)	0.73 (0.10)
multilingual-E5 + eGeMAPS	1024 + 88	0.64 (0.05)	0.70 (0.09)
multilingual-E5 + audEERING wav2vec2	1024 + 1027	0.64 (0.04)	0.72 (0.10)
multilingual-E5 + ComParE	1024 + 6373	0.63 (0.07)	0.69 (0.12)

Abbreviations: bACC, balanced accuracy; AUC-ROC, area under the ROC curve; SD, standard deviation.

Supplementary Table 5: Pairwise effect size comparisons between models for the HC vs. MDD classification analysis. Effect sizes are computed on balanced accuracy. Δ is defined as B−A (Feature Set B minus A); higher is better, so negative Δ favors the first model (A). Cohen's d_z is computed on outer-fold differences; 95% CIs from 10,000 bootstrap iterations.

**Comparison**	**Δ bACC (95% CI)**	**Cohen's d_z (95% CI)**	**Cliff's Δ**
Qwen3-8B vs			
multilingual-E5 + Qwen3-8B	0.00 (-0.04, 0.04)	0.06 (-1.45, 1.61)	-0.20
Qwen3-8B + audEERING	-0.03 (-0.09, 0.01)	-0.47 (-1.54, 0.56)	-0.20
TF–IDF	-0.07 (-0.13, -0.01)	-0.91 (-13.50, -0.08)	-0.60
Timing & Lexical	-0.09 (-0.19, 0.00)	-0.78 (-6.75, 0.05)	-0.20
audEERING wav2vec2	-0.13 (-0.24, -0.02)	-0.94 (-2.66, -0.44)	-0.60
eGeMAPS	-0.16 (-0.28, -0.06)	-1.19 (-3.29, -0.89)	-1.00
ComParE	-0.14 (-0.26, -0.02)	-0.90 (-2.63, -0.21)	-0.60

Abbreviations: bACC, balanced accuracy; CI, confidence interval; d_z, Cohen's d_z.

Supplementary Table 6: Predictive performance within MDD cohort only (N=128). All models are Support Vector Regressors with hyperparameters tuned via 5×3 nested cross-validation (5 outer, 3 inner folds). Best single modality and best multimodal combination are bold.

Feature Set	k (features)	MAE (SD)	R^2 (SD)
Any Feature Set (Dummy Baseline)	—	7.37 (1.60)	-0.06 (0.19)
**Single Modality**			
**multilingual-E5**	**1024**	**6.74 (1.42)**	**0.20 (0.05)**
Qwen3-8B	4096	6.86 (1.38)	0.17 (0.08)
Qwen3-4B	2560	6.93 (1.45)	0.16 (0.09)
TF–IDF	variable	6.98 (1.28)	0.10 (0.08)
Qwen3-0.6B	1024	7.14 (1.53)	0.12 (0.03)
audEERING wav2vec2	1027	7.22 (1.01)	0.02 (0.14)
Facebook HuBERT-Base	768	7.31 (1.39)	0.00 (0.05)
All-MPNet-Base-v2	768	7.33 (1.66)	0.03 (0.06)
EmbeddingGemma	768	7.33 (1.82)	0.07 (0.09)
Microsoft WavLM-Base	768	7.40 (1.32)	0.00 (0.01)
ComParE	6373	7.46 (1.20)	-0.01 (0.03)
eGeMAPS	88	7.47 (1.30)	-0.02 (0.02)
Facebook wav2vec 2.0 Base	768	7.56 (1.13)	-0.06 (0.14)
Timing & Lexical features	18	7.71 (0.99)	-0.13 (0.15)
**Multimodal Combinations**			
**multilingual-E5 + audEERING wav2vec2**	**1024 + 1027**	**6.08 (1.59)**	**0.22 (0.09)**
multilingual-E5 + Timing & Lexical features	1024 + 18	6.17 (1.64)	0.20 (0.08)
multilingual-E5 + eGeMAPS	1024 + 88	6.22 (1.62)	0.20 (0.07)
multilingual-E5 + ComParE	1024 + 6373	6.20 (1.60)	0.19 (0.07)
multilingual-E5 + TF–IDF	1024 + variable	6.24 (1.61)	0.19 (0.04)
multilingual-E5 + Qwen3-8B	1024 + 4096	6.24 (1.60)	0.19 (0.10)
Qwen3-8B + audEERING wav2vec2	4096 + 1027	6.31 (1.67)	0.17 (0.15)
Qwen3-8B + Timing & Lexical features	4096 + 18	6.47 (1.60)	0.14 (0.12)
Qwen3-8B + TF–IDF	4096 + variable	6.50 (1.63)	0.13 (0.10)
Qwen3-8B + eGeMAPS	4096 + 88	6.53 (1.59)	0.13 (0.11)
Qwen3-8B + ComParE	4096 + 6373	6.50 (1.59)	0.13 (0.12)

Abbreviations: MAE, mean absolute error; R^2, coefficient of determination; SD, standard deviation.

Supplementary Table 7: Pairwise effect size comparisons between models for the MDD-only regression analysis. Effect sizes are based on R^2. Δ is defined as B$-$A (Feature Set B minus A); higher is better, so negative Δ favors the first model (A). Cohen's d_z is computed on outer-fold differences; 95% CIs from 10,000 bootstrap iterations.

**Comparison**	**ΔR^2 (95% CI)**	**Cohen's d_z (95% CI)**	**Cliff's Δ**
Qwen3-8B vs			
TF–IDF	-0.07 (-0.16, 0.03)	-0.59 (-4.41, 0.29)	-0.60
Timing & Lexical	-0.30 (-0.43, -0.20)	-2.06 (-8.84, -1.72)	-1.00
audEERING wav2vec2	-0.15 (-0.21, -0.07)	-1.67 (-15.48, -0.83)	-1.00
eGeMAPS	-0.19 (-0.24, -0.12)	-2.42 (-9.58, -1.40)	-1.00
ComParE	-0.18 (-0.23, -0.13)	-2.93 (-19.43, -1.99)	-1.00
multilingual-E5 + Qwen3-8B	0.01 (-0.08, 0.10)	0.12 (-1.27, 1.87)	0.20
Qwen3-8B + audEERING	0.00 (-0.15, 0.14)	0.00 (-1.30, 1.19)	-0.20

Abbreviations: CI, confidence interval; ΔR^2, difference in R^2 (B minus A); d_z, Cohen's d_z.

Supplementary Table 8: Predictive performance within HC cohort only (N=101). All models are Support Vector Regressors with hyperparameters tuned via 5×3 nested cross-validation (5 outer, 3 inner folds). Best single modality and best multimodal combination are bold.

Feature Set	k (features)	MAE (SD)	R^2 (SD)
**Single Modality**			
**Qwen3-8B**	**4096**	**2.87 (1.05)**	**-0.08 (0.22)**
Qwen3-4B	2560	2.89 (1.06)	-0.10 (0.21)
multilingual-E5	1024	2.88 (1.04)	-0.11 (0.34)
Timing & Lexical features	18	2.90 (1.38)	-0.15 (0.14)
Microsoft WavLM-Base	768	2.98 (1.19)	-0.18 (0.15)
Qwen3-0.6B	1024	2.97 (1.17)	-0.18 (0.22)
eGeMAPS	88	3.02 (1.25)	-0.18 (0.20)
Facebook HuBERT-Base	768	3.01 (1.27)	-0.21 (0.16)
TF–IDF	variable	3.06 (1.26)	-0.22 (0.13)
EmbeddingGemma	768	3.04 (1.20)	-0.22 (0.26)
audEERING wav2vec2	1027	3.02 (1.28)	-0.23 (0.18)
Facebook wav2vec 2.0 Base	768	3.02 (1.23)	-0.24 (0.25)
ComParE	6373	3.04 (1.26)	-0.25 (0.25)
All-MPNet-Base-v2	768	3.10 (1.25)	-0.25 (0.24)
**Multimodal Combinations**			
**multilingual-E5 + eGeMAPS**	**1024 + 88**	**3.21 (0.97)**	**-0.44 (0.55)**
multilingual-E5 + Timing & Lexical features	1024 + 18	3.25 (1.05)	-0.44 (0.57)
multilingual-E5 + TF–IDF	1024 + variable	3.28 (1.06)	-0.47 (0.58)
Qwen3-8B + eGeMAPS	4096 + 88	3.21 (0.90)	-0.48 (0.66)
Qwen3-8B + Timing & Lexical features	4096 + 18	3.26 (1.04)	-0.48 (0.67)
multilingual-E5 + ComParE	1024 + 6373	3.22 (0.98)	-0.52 (0.63)
Qwen3-8B + ComParE	4096 + 6373	3.17 (0.96)	-0.52 (0.72)
Qwen3-8B + TF–IDF	4096 + variable	3.27 (0.98)	-0.53 (0.71)
Qwen3-8B + audEERING wav2vec2	4096 + 1027	3.57 (1.12)	-0.68 (0.78)
multilingual-E5 + audEERING wav2vec2	1024 + 1027	3.64 (1.06)	-0.76 (0.68)
multilingual-E5 + Qwen3-8B	1024 + 4096	3.50 (1.08)	-1.03 (1.49)

Abbreviations: MAE, mean absolute error; R^2, coefficient of determination; SD, standard deviation.

Supplementary Table 9: Pairwise effect size comparisons between models for the HC-only regression analysis. Effect sizes are based on R^2. Δ is defined as B−A (Feature Set B minus A); higher is better, so positive Δ favors the first model (A). Cohen's d_z is computed on outer-fold differences; 95% CIs from 10,000 bootstrap iterations. Note: All models showed poor performance in the HC cohort (negative R^2 values).

**Comparison**	**ΔR^2 (95% CI)**	**Cohen's d_z (95% CI)**	**Cliff's Δ**
Qwen3-8B vs			
TF–IDF	0.14 (0.05, 0.36)	0.63 (-0.38, 3.73)	0.60
Timing & Lexical	0.07 (-0.01, 0.32)	0.52 (-0.07, 2.96)	0.40
audEERING wav2vec2	0.15 (0.01, 0.45)	0.84 (-0.06, 5.00)	0.80
eGeMAPS	0.10 (-0.04, 0.43)	0.50 (-0.20, 2.51)	0.60
ComParE	0.17 (-0.01, 0.55)	0.69 (-0.04, 3.85)	0.80
multilingual-E5 + Qwen3-8B	0.95 (-0.81, 2.88)	0.64 (-0.55, 1.94)	0.60
Qwen3-8B + audEERING	0.60 (-0.29, 1.65)	0.77 (-0.37, 2.12)	0.80

Abbreviations: CI, confidence interval; d_z, Cohen's d_z.

Supplementary Table 10: Error distribution overall and by BDI band for the best model. Columns report mean absolute error (MAE) and the percentage of predictions within absolute-error thresholds.

	**MAE**	≤ 3	≤ 5	≤ 7	≤ 10
Overall	4.52	45.9	68.2	81.1	91.5
BDI 0–13	3.46	52.0	76.5	89.1	97.6
BDI 14–19	6.24	23.8	40.1	59.6	83.4
BDI 20–28	10.63	10.6	18.6	28.0	44.7
BDI $\geq$29	22.24	0.0	1.4	4.3	10.0

Abbreviations: MAE, mean absolute error.

Supplementary Table 11: Performance degradation under controlled linguistic perturbations testing contributions of syntax, morphology, and subword information. Performance under linguistic perturbations (means ± SD across outer folds). Δ values and p-values are paired permutation tests vs. baseline (out-of-sample predictions; $n = 3165$ pairs).

**Condition**	**MAE**	R^2	**Δ**MAE	ΔR^2
Baseline	4.56 ± 0.55	0.33 ± 0.03	—	—
P1: Word-order disruption	4.83 ± 0.56	0.23 ± 0.05	$+0.267$ (5×10^{-4})	-0.08 (5×10^{-4})
P2: Inflection neutralization (lemmatize)	4.61 ± 0.58	0.30 ± 0.03	$+0.049$ (0.082)	-0.02 (0.0020)
P3: Function-word skeleton (drop content)	5.13 ± 0.63	0.11 ± 0.17	$+0.758$ (5×10^{-4})	-0.25 (5×10^{-4})
P3: Content-only (drop function words)	4.87 ± 0.43	0.23 ± 0.04	$+0.286$ (5×10^{-4})	-0.08 (5×10^{-4})

Abbreviations: MAE, mean absolute error; R^2, coefficient of determination; Δ, difference to baseline; SD, standard deviation. p-values are from paired permutation tests vs. baseline (outer-fold out-of-sample predictions).
